# Selection and Validation of Reference Genes for RT-qPCR Analysis of the Ladybird Beetle *Henosepilachna vigintioctomaculata*

**DOI:** 10.3389/fphys.2018.01614

**Published:** 2018-11-14

**Authors:** Jing Lü, Shimin Chen, Mujuan Guo, Cuiyi Ye, Baoli Qiu, Jianhui Wu, Chunxiao Yang, Huipeng Pan

**Affiliations:** ^1^Key Laboratory of Bio-Pesticide Innovation and Application of Guangdong Province, Department of Entomology, South China Agricultural University, Guangzhou, China; ^2^State Key Laboratory for Conservation and Utilization of Subtropical Agro-Bioresources, South China Agricultural University, Guangzhou, China

**Keywords:** *Henosepilachna vigintioctopunctata*, RT-qPCR analysis, reference gene, *RefFinder*, *geNorm*

## Abstract

Reverse transcriptase-quantitative polymerase chain reaction (RT-qPCR) is a momentous technique for quantifying expression levels of the targeted genes across various biological processes. Selection and validation of appropriate reference genes for RT-qPCR analysis are a pivotal precondition for reliable expression measurement. *Henosepilachna vigintioctopunctata* is one of the most serious insect pests that attack Solanaceae plants in Asian countries. Recently, the transcriptomes of *H. vigintioctopunctata* were sequenced, promoting gene functional studies of this insect pest. Unfortunately, the reference genes for *H. vigintioctopunctata* have not been selected and validated. Here, a total of 7 commonly used reference genes, namely, *Actin*, *GAPDH*, *RPL13*, *RPL6*, *RPL32*, *RPS18*, and *ATPB*, were selected and assessed for suitability under four experimental conditions, namely, developmental stage, tissue, temperature, and host plant, using *RefFinder*, which integrates four different analytical tools (*Normfinder*, *geNorm*, the Δ*Ct* method, and *BestKeeper*). The results displayed that *RPL13* and *RPS18* were the best suitable reference genes for each experimental condition. The relative transcript levels of 2 target genes, *lov* and *TBX1*, varied greatly according to normalization with the two most- and least-suited reference genes. Our results will be helpful for improving the accuracy of the RT-qPCR analysis for future functional investigations of target gene expression in *H. vigintioctopunctata*.

## Introduction

Reverse transcriptase-quantitative polymerase chain reaction (RT-qPCR) is a frequently used technique for gene expression studies on account of its high specificity, high sensitivity, high throughput, and low cost (Hellemans and Vandesompele, 2014). However, many factors relevant to biological and technical variations, for instances, RNA isolation, integrity, purity; reverse transcription; PCR efficiency, can affect the precision of RT-qPCR analysis (Bustin et al., 2005, 2009). Generally, RT-qPCR involves standardization to the expression of a battery of appropriately stable reference genes concurrent. In spite of reference gene transcript levels should be stably expressed in a serious of different biological or experimental conditions, previous studies have shown that many frequently used reference genes differ observably in different treatments (Li et al., 2013; Yang et al., 2014, 2015a,b,c, 2016, 2018; Pan et al., 2015a,b). Therefore, a systematic and customized study for each tested species is recommended for identifying appropriate reference genes.

*Henosepilachna vigintioctopunctata* (Fabricius) (Coleoptera: Coccinellidae) is one of the most serious insect pests in Asian countries (Ghosh and Senapati, 2001; Shinogi et al., 2005; Venkatesha, 2006; Zhou et al., 2015). *H. vigintioctopunctata* colonizes many different species of plants, for example, solanaceous plants such as eggplant, tomato, potato, and pepper; cucurbitaceous plants such as cucumber, white gourd, and loofah. In addition, it attacks many weeds, such as the black nightshade, winter cherry, thorn apple, and tobacco (Pang and Mao, 1979). The destructive potential of *H. vigintioctopunctata* is high at both the adult and larval stages, leading to up to 60% loss of fruit production (Sharma et al., 2012; Kawazu, 2014).

In China, *H. vigintioctopunctata* is widely distributed from Hainan Province in the south to Heilongjiang Province in the north and from Gansu Province in the west to Shanghai City in the east (Pang and Mao, 1979). Recently, because of climate warming, development of trade, and expansion of the cultivated area for protected vegetables, the food for *H. vigintioctopunctata* is constantly increasing throughout the year, and the occurrence and damage of *H. vigintioctopunctata* have become significant (Li et al., 2006; Zhou et al., 2015; Wang et al., 2017). Recently, we sequenced the transcriptomes of *H. vigintioctopunctata* at different developmental stages (unpublished data) and obtained a huge amount of genes involved in the development, energy metabolism, and reproduction. Recently, RNA interference (RNAi) has been used widely in the study of functional genomics, resulting in the development of new modes of action insecticides for insect pest management (Baum et al., 2007; Bellés, 2010; Burand and Hunter, 2013; Scott et al., 2013; Zhang et al., 2017). To identify novel target genes for controlling *H. vigintioctopunctata*, accurate gene expression of this pest under different biotic and abiotic conditions is necessary.

In this study, to discern the solidly expressed reference genes of *H. vigintioctopunctata* for RT-qPCR investigation under four experimental conditions (developmental stage, tissue, temperature, and host plant), 7 most commonly used reference genes, namely, actin (*Actin*), ribosomal protein L13 (*RPL13*), glyceraldehyde 3-phosphate dehydrogenase (*GAPDH*), ribosomal protein L6 (*RPL6*), ribosomal protein L32 (*RPL32*), ribosomal protein S18 (*RPS18*), and vacuolar-type H^+^-ATPase subunit B (*ATPB*), were selected from the transcriptomes of *H. vigintioctopunctata*. The expression stability of each candidate reference gene was assessed under 2 biotic (developmental stage and tissue) and 2 abiotic (temperature and host plant) conditions by using *RefFinder*, which integrates four different analytical tools (*Normfinder*, *geNorm*, the Δ*Ct* method, and *BestKeeper*). Finally, jim lovell (*lov*) and T-box transcription factor (*TBX1*) were used as the target genes to verify our findings. The results will be useful in improving the accuracy of RT-qPCR analysis for future functional investigations of target gene expression in *H. vigintioctopunctata*.

## Materials and Methods

### Insects

In April 2018, the adults of *H. vigintioctopunctata* were collected from *Solanum nigrum* (L.) in Guangzhou City, Guangdong Province, China, and reared in the incubator at 25 ± 0.5°C temperature, 14L:10D photoperiod, and 80% relative humidity in petri dishes by using *S. nigrum* and *S. melongena* leaves.

### Sample Treatment and Collection

#### Biotic Factors

All stages of *H. vigintioctopunctata* were sampled: eggs, four larval instars, pupae, and female and male adults (collected on the first day of each stage). The number of sampled individuals for each replicate across the different developmental stage was as follows: 20 eggs for the egg stage; 10 individuals for the first instar; 5 individuals for the second instar; 3 individuals for the third instar; 1 individual for the fourth instar; 1 pupa for the pupal stage; and 1 male or female individual for the adult male or female stage. Different body tissues, namely, the Malpighian tubule, fat body, midgut, and cuticle, were dissected from the fourth instar larvae, about 40 individuals were dissected for each replicate. The tissue samples were placed in RNA*later*^®^ (Thermo Fisher Scientific Inc., United Stats) and stored at 4°C before total RNA isolation.

#### Abiotic Factors

For the temperature treatment, 5 s instars were exposed to 8, 25, and 35°C for 3 h. For the host plant treatment, 2 third instars larvae were collected as one sample that reared on *S. nigrum* and eggplant.

Each experiment was replicated 3 times. All the samples, except the ones used for the host plant treatment, were collected from the colony reared on *S. nigrum*. All the samples, except the tissue ones, were placed in 1.5 ml RNA free centrifuge tubes, rapidly frozen in liquid nitrogen, and stored at -80°C before the total RNA extraction.

### Total RNA Extraction and cDNA Synthesis

The total RNAs of egg, Malpighian tubule, and fat body were isolated using TRIzol reagent (Invitrogen, United States), in line with our previously described methods (Pan et al., 2015a). Total RNAs from the other samples were isolated using a HiPure Total RNA Micro Kit (Magen, China), according to the manufacturer’s instructions. A NanoDrop One^C^ spectrophotometer (Thermo Fisher Scientific, Waltham, MA United States) was used to ascertain the RNA concentration. The total RNA was dissolved in 20–100 μl of ddH_2_O, and the concentrations were as follows: 494.3 ± 16.7 ng/μl [mean ± standard error of the mean (SEM)] for the eggs, 427.6 ± 28.6 ng/μl for the first instars, 683.9 ± 79.8 ng/μl for the second instars, 567.1 ± 38.7 ng/μl for the third instars, 619.8 ± 61.9 ng/μl for the fourth instars, 818.6 ± 59.1 ng/μl for the pupae, 760.2 ± 36.6 ng/μl for the male adults, 763.4 ± 78.9 ng/μl for the female adults, 677.5 ± 68.0 ng/μl for the cuticles, 565.3 ± 38.1 ng/μl for the fat body, 870.3 ± 22.6 ng/μl for the midguts, 483.8 ± 74.9 ng/μl for the Malpighian tubules, 468.4 ± 6.8 ng/μl for the second-instar under 15°C, 683.9 ± 79.8 ng/μl for the second-instar under 25°C, 356.1 ± 7.9 ng/μl for the second-instar under 35°C, and 422.1 ± 68.4 ng/μl for the third-instar under eggplant. The OD260/280 value of all samples was 1.9–2.1. The PrimeScript RT kit (containing gDNA Eraser, Perfect Real Time, TaKaRa, China) was used for preparing the first-strand cDNA for gene expression investigation. The cDNA was diluted 10-fold for the pursuant RT-qPCR experiments.

### Primer Design and Gene Cloning

In our study, 7 candidate reference genes that are most frequently used in RT-qPCR investigations were assessed (Table 1). The primers were designed based on the PrimerQuest Tool^1^, according to the sequences obtained from our recently sequenced transcriptomes for *H. vigintioctopunctata* (unpublished data).

**Table 1:** Reference genes used in this study.

Gene	Primer sequences (5′–3′)	Length (bp)	Efficiency (%)	*R*^2^	Linear regression
*Actin*	F: TCGTGACTTGACTGACTACCT	128	93.00	0.9986	y = -3.5019x+21.165
	R: GCTCGAAGTCCAAAGCTACAT				
*GAPDH*	F: AGCTCTTCTCATCATGGCTTAC	127	91.23	0.9945	y = -3. 5518x+22.019
	R: GAAAGAGGTGCAGAATGTGTTG				
*RPL13*	F: AGCATCCTTCGCTCGTTTAG	137	92.55	0.995	y = -3.5144x+20.734
	R: TTCGACAACCTGCCATTAGG				
*RPL6*	F: CACTGGACCTTTCGGTATCAA	142	91.86	0.997	y = -3.5337x+29.152
	R:TTCCCTCACTCTCCTGAAGTA				
*RPL32*	F: TATGGGCTGTACCCAAACAC	125	100.62	0.9989	y = -3.3071x+28.81
	R: GCCACATGTATTGCAGATTCG				
*RPS18*	F: CGCAATCAAAGGTGTTGGAAG	134	94.61	0.9952	y = -3.4583x+20.263
	R: GCCTAGGGTTGGCCATAATAG				
*ATPB*	F:CCCATCCCATTCCTGATTTGA	119	97.28	0.9949	y = -3.389x+22.735
	R: CAAACGAGACAGAGACGGTAAT				

The PCR reaction system and parameters were used according to our previous study (Yang et al., 2014). Amplicons of the expected lengths were purified using the TIANgel Midi Purification Kit (Tiangen, China), and subcloned into the pClone007 Blunt vector before transformation into *Escherichia coli* DH5α competent cells (Tsingke, China) for sequencing by Tsingke company. The reference genes were confirmed using sequence analysis.

### RT-qPCR Analysis

The RT-qPCR reactions and program were conducted, the melting curve and standard curve for each reference gene were generated according to our previous study (Yang et al., 2014, 2015a,b,c, 2016, 2018). The homologous RT-qPCR efficiencies (E) were calculated according to the equation: E = (10^[-1/slope]^ -1) × 100.

### Determination the Expression Stability of Reference Genes

The reference gene expression stability was assessed using *RefFinder*^2^, which integrates four different analytical tools, *NormFinder* (Andersen et al., 2004), *geNorm* (Vandesompele et al., 2002), the Δ*C_t_* method (Silver et al., 2006), and *BestKeeper* (Pfaffl et al., 2004). The optimal number of reference genes for target gene expression normalization was decided by pairwise variation (V_n_/V_n+1_). A V_n_/V_n+1_ cutoff value of 0.15 indicates the additional 1 more reference gene is not necessary, i.e., the starting n reference genes are enough for the target gene normalization; the V-values were calculated using *geNorm* (Vandesompele et al., 2002).

### Determination of Gene Expression Levels on the Basis of Different Reference Genes

*Lov* is a nuclear protein has roles in various larval and adult behaviors (Bjorum et al., 2013). *TBX1* plays a vital role in the upgrowth of *Drosophila* heart (Griffin et al., 2000). The stability of these reference genes was investigated using *lov* and *TBX1* as the target genes. The primer sequences of these two target genes were as follows: *lov*, forward (5′-CTCCCGCCCAACACTTTAT-3′) and reverse (5′-TCGCTTTGCGGTAGTAGATG-3′); *TBX1*, forward (5′-GAAACACCTCTGGGACGAAT-3′) and reverse (5′-TCGGAGTGCAAGTCTAAACC-3′). *Lov* and *TBX1* expression levels in different tissues were computed on the basis of normalization to the 2 most- and 2 least-stable candidates. The relative gene expression of *lov* and *TBX1*was computed using the 2^-ΔΔCt^ method (Livak and Schmittgen, 2001). One-way analysis of variance was used to detect significances in *lov* and *TBX1* expression levels among different tissues (SPSS 17.0).

## Results

### Reference Gene Expression Profiles

**FIGURE 1:**
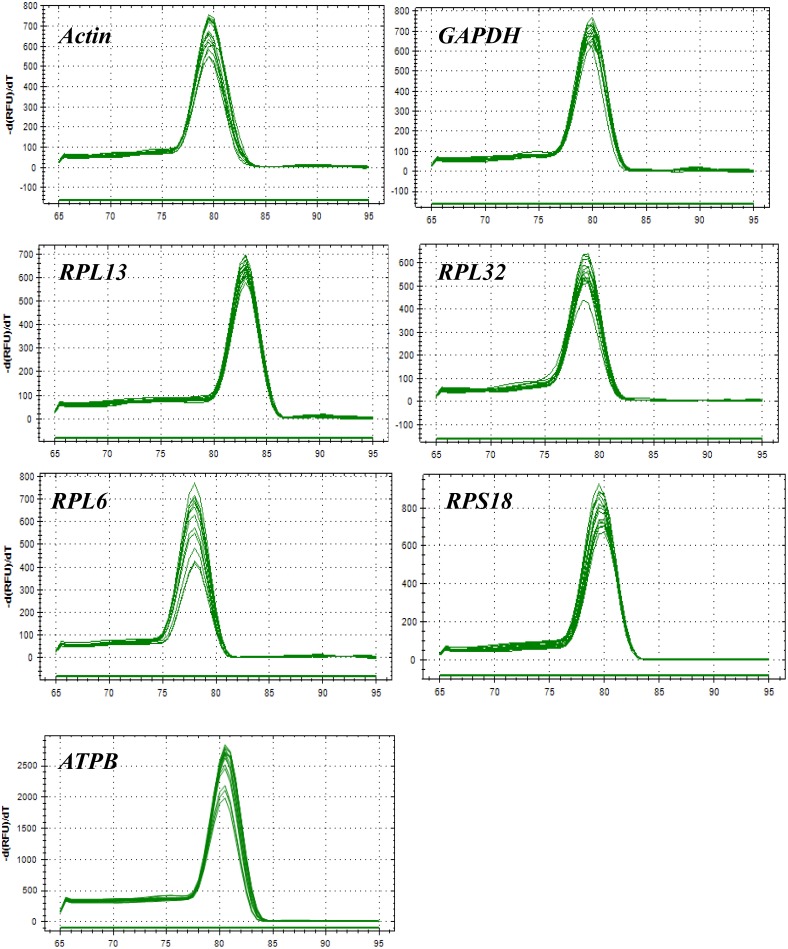
Melting curves of the 7 candidate reference genes in *H. vigintioctomaculata*.

All of these candidate reference genes were expressed in *H. vigintioctopunctata* and intuitional with a single amplicon of the expected size for each gene (Supplementary Figure S1). Gene-specific amplification of all reference genes was verified by a single peak in the melting curve analysis (Figure 1). The PCR efficiency ranged between 92 and 100% (Table 1). The standard curve for each reference gene is also provided (Supplementary Figure S2).

**FIGURE 2:**
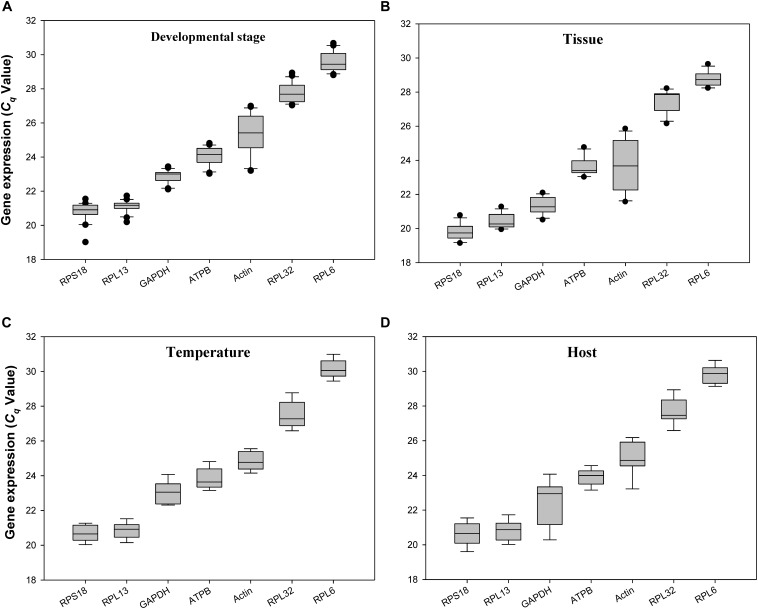
Expression profiles of the 7 candidate reference genes in all 4 experiments for *H. vigintioctomaculata*. The expression levels of the reference genes are shown in terms of the *C_q_*-value for each experimental condition. **(A)** Development stage, **(B)** Tissue, **(C)** Temperature, **(D)** Host plant.

The quantification cycle (*C_q_*) values for all the reference genes under the 4 experimental conditions ranged from 20 to 29. *RPS18* and *RPL13* were the most abundant reference genes, whereas *RPL32* and *RPL6* were the least expressed ones (Figure 2).

### Stability of the Reference Genes Under Different Experimental Conditions

**FIGURE 3:**
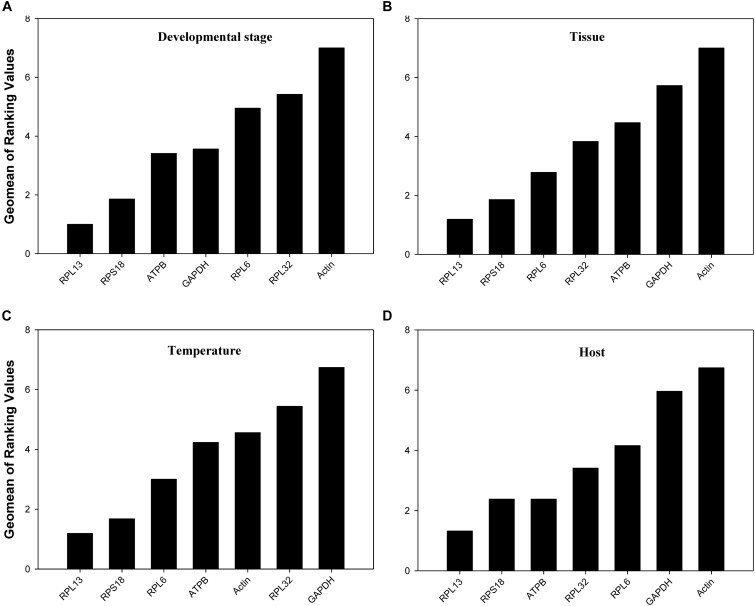
Stability of the 7 candidate reference gene expressions in *H. vigintioctomaculata* under different treatment conditions analyzed using *RefFinder*. **(A)** Development stage, **(B)** Tissue, **(C)** Temperature, **(D)** Host plant.

According to *RefFinder*, across different development stages, the comprehensive reference gene rankings from the best to the least stable were as follows: *RPL13*, *RPS18*, *ATPB*, *GAPDH*, *RPL6*, *RPL32*, and *Actin* (Figure 3A). For the tissue treatment, the comprehensive reference gene rankings were as follows: *RPL13*, *RPS18*, *RPL6*, *RPL32*, *ATPB*, *GAPDH*, and *Actin* (Figure 3B). Under different temperature conditions, the integrated reference gene rankings were as follows: *RPL13*, *RPS18*, *RPL6*, *ATPB*, *Actin*, *RPL32*, and *GAPDH* (Figure 3C). For the host plant treatment, the comprehensive reference gene rankings were as follows: *RPL13*, *RPS18*, *ATPB*, *RPL32*, *RPL6*, *GAPDH*, and *Actin* (Figure 3D). The expression stability value for each gene was also computed using *Normfinder*, *geNorm*, the Δ*Ct* method, and *BestKeeper* under each experimental condition (Table 2).

**Table 2:** Stability of 7 candidate reference gene expression in *H. vigintioctomaculata* under different experimental conditions calculated by the 4 different analytical tools *geNorm*, *Normfinder*, *BestKeeper*, and the Δ*Ct* method, respectively.

Conditions	CRGs^∗^	*geNorm*		*NormFinder*		*BestKeeper*		Δ*Ct*		Recommendation
		Stability	Rank	Stability	Rank	Stability	Rank	Stability	Rank	
Developmental stage	*RPL13*	0.338	1	0.169	1	0.28	1	0.54	1	
	*RPS18*	0.338	1	0.283	2	0.40	3	0.62	2	
	*ATPB*	0.427	2	0.407	3	0.49	5	0.67	3	
	*GAPDH*	0.543	4	0.475	4	0.31	2	0.72	4	*RPL13*, *RPS18*
	*RPL6*	0.575	5	0.513	5	0.47	4	0.73	5	
	*RPL32*	0.492	3	0.580	6	0.55	6	0.75	6	
	*Actin*	0.741	6	1.083	7	0.97	7	1.16	7	
Tissue	*RPL13*	0.348	1	0.185	1	0.34	1	0.67	1	
	*RPS18*	0.348	1	0.317	2	0.34	1	0.70	2	
	*ATPB*	0.611	4	0.460	4	0.41	2	0.82	5	
	*GAPDH*	0.672	5	0.804	6	0.42	3	0.95	6	*RPL13*, *RPS18*
	*RPL6*	0.426	2	0.535	5	0.34	1	0.79	4	
	*RPL32*	0.539	3	0.398	3	0.54	4	0.78	3	
	*Actin*	0.866	6	1.284	7	1.14	5	1.35	7	
Temperature	*RPL13*	0.185	1	0.093	1	0.34	1	0.44	2	
	*RPS18*	0.185	1	0.093	2	0.42	3	0.43	1	
	*ATPB*	0.281	3	0.316	4	0.51	4	0.50	4	
	*GAPDH*	0.578	6	0.774	7	0.53	5	0.85	7	*RPL13*, *RPS18*
	*RPL6*	0.265	2	0.216	3	0.41	2	0.47	3	
	*RPL32*	0.336	4	0.462	5	0.61	6	0.59	5	
	*Actin*	0.470	5	0.655	6	0.41	2	0.76	6	
Host plant	*RPL13*	0.133	1	0.067	1	0.53	2	0.62	1	
	*RPS18*	0.133	1	0.252	4	0.59	3	0.66	2	
	*ATPB*	0.327	3	0.103	2	0.37	1	0.71	4	
	*GAPDH*	0.469	4	0.948	6	1.10	6	1.03	6	*RPL13*, *RPS18*
	*RPL6*	0.589	5	0.507	5	0.53	2	0.90	5	
	*RPL32*	0.237	2	0.205	3	0.60	4	0.69	3	
	*Actin*	0.900	6	1.639	7	0.87	5	1.68	7	

*^∗^Candidate reference gene.*

### Recommended Reference Genes Depended on *geNorm*

**FIGURE 4:**
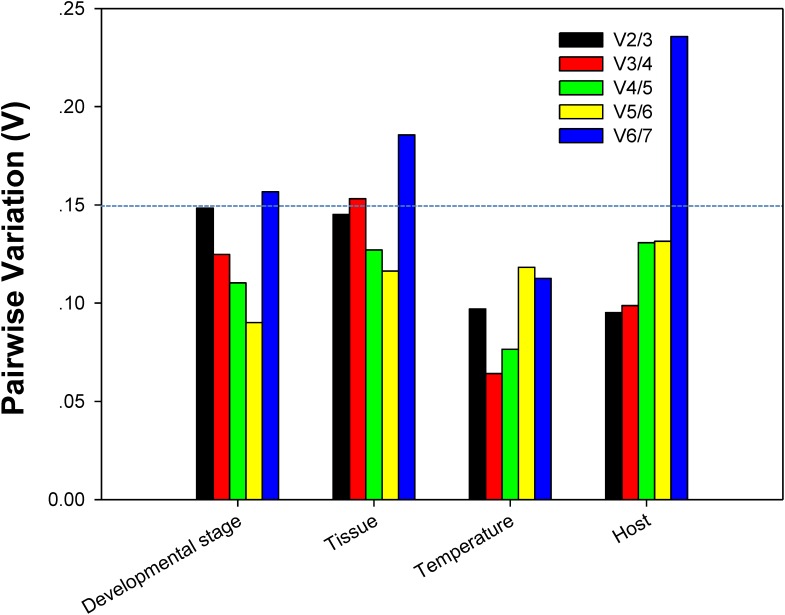
Optimum number of reference genes required for accurate normalization of gene expression. Pairwise variation (V) values in the 4 groups using *geNorm*.

For each of four different experimental conditions, the initial *V*-value < 0.15 were all emerged at V2/3, respectively, suggesting 2 reference genes were enough for the target gene normalization. By coincidence, *RPL13* and *RPS18* were the best stable reference genes for each experimental condition, respectively (Figure 4).

### Validation of the Selected Reference Genes

**FIGURE 5:**
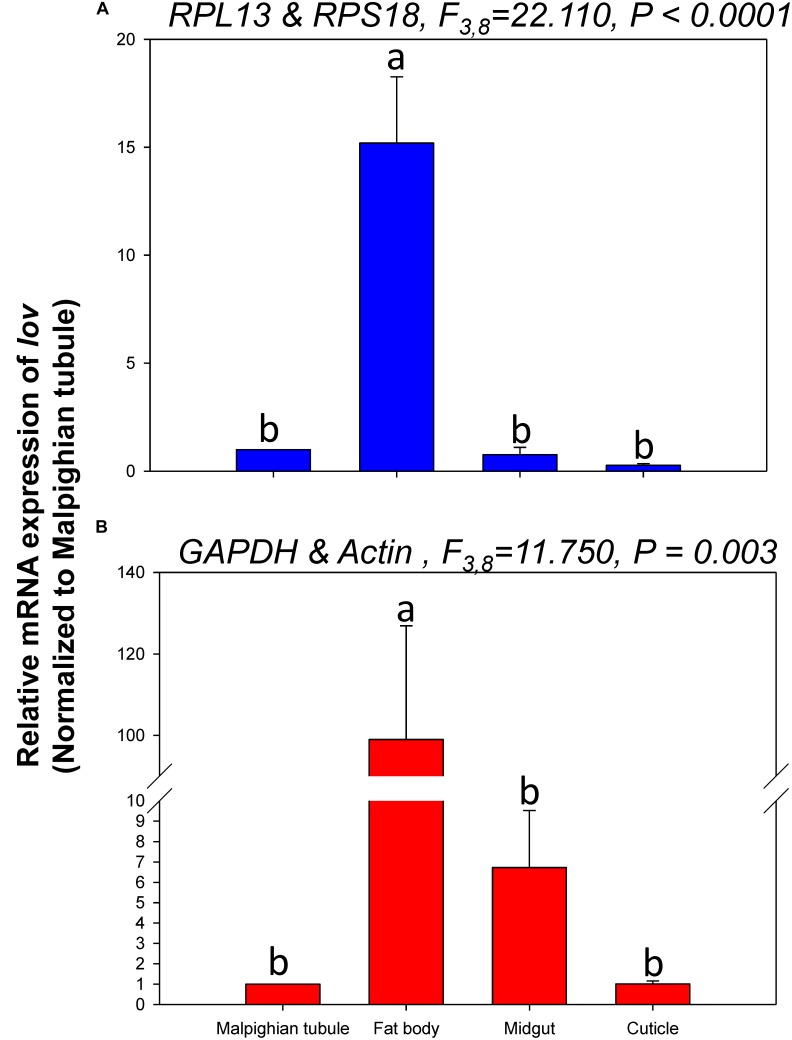
Relative gene expression of *lov* in different tissues of *H. vigintioctomaculata*. The relative abundance of *lov* in the Malpighian tubule, fat body, midgut, and cuticle were normalized to the best stable (**A**, *RPL13* and *RPS18*) and least stable (**B**, *GAPDH* and *Actin*) reference genes, respectively. The values are means + SE. Different letters indicate significant differences in gene expression among different tissues of *H. vigintioctomaculata* (*P* < 0.05).

**FIGURE 6:**
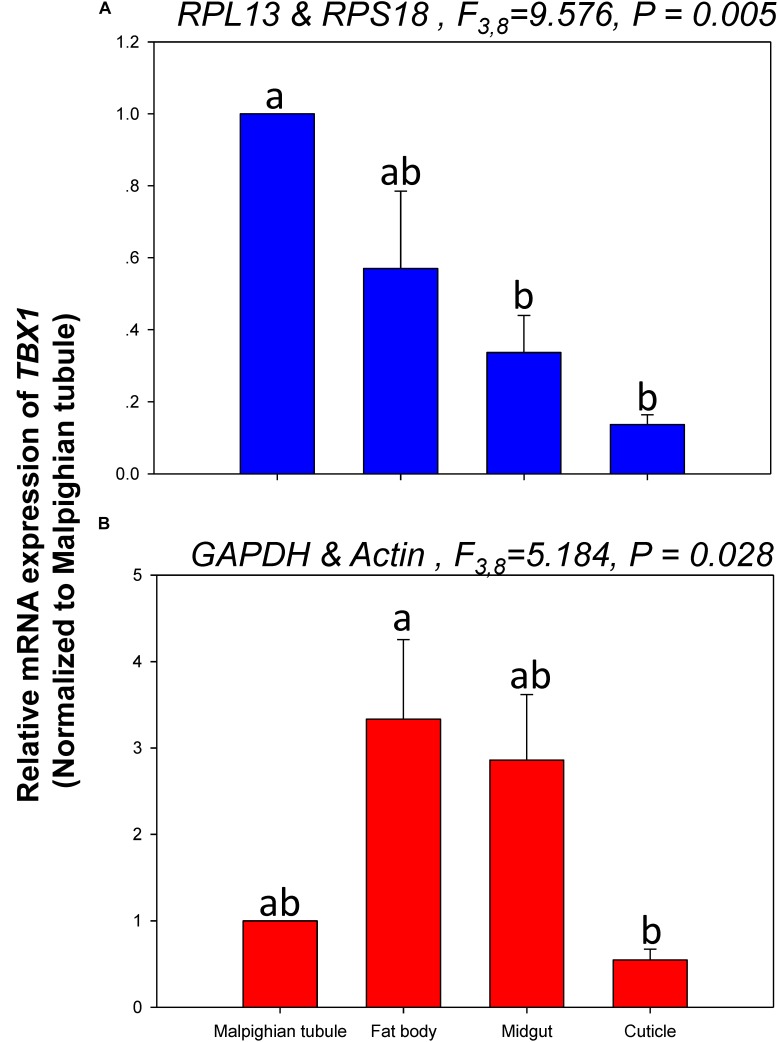
Relative gene expression of *TBX1* in different tissues of *H. vigintioctomaculata*. The relative gene expression levels of *TBX1* in the Malpighian tubule, fat body, midgut, and cuticle were normalized to the best stable (**A**, *RPL13* and *RPS18*) and least stable (**B**, *GAPDH* and *Actin*) reference genes, respectively. The values are means + SE. Different letters indicate significant differences in gene expression among different tissues of *H. vigintioctomaculata* (*P* < 0.05).

The relative expression levels of *lov* and *TBX1* were used to validate the reference genes among the different tissues. The *Lov* expression patterns were similar, however, *lov* expression was about 15- and 99-fold higher in the fat body than in the Malpighian tubule when normalized to the 2 most- and least-stable reference genes, respectively (Figure 5). In contrast, *TBX1* expression patterns were inconsistent among the different tissues when normalized to the 2 most- and least-stable reference genes. Different degrees of gene expression were obtained in each tissue under the 2 normalization conditions (Figure 6).

## Discussion

Previous studies have demonstrated there is no “universal” reference gene applicable for various test conditions, even for the same insect species. For example, five papers have been published for the reference gene selection of the whitefly, *Bemisia tabaci*, a notorious and invasive insect species, in the past 5 years (Li et al., 2013; Su et al., 2013; Collins et al., 2014; Liang et al., 2014; Dai et al., 2017). It is pivotal to select and validate the reference genes expression stability under various experimental conditions precedence using them for normalizing gene expression.

We sequenced the transcriptomes for the developmental stages of *H. vigintioctopunctata* (unpublished data). In the future, we will use RNA interference to investigate the gene functions, and RT-qPCR will be widely applied for evaluating the gene expression changes. However, the reference genes have not been previously selected and validated in this insect pest. Therefore, in this study, seven frequently used reference genes were picked out and their stability was investigated using five software programs under four experimental conditions. The results displayed that the reference gene transcript levels vary with the experimental conditions. Thus, it is no doubt that the expression profiles of the target genes will show substantive variations relying on the reference gene and experimental treatments (Yang et al., 2016).

Our results certified that 5 computational methods yielded disparate stability rankings for the seven reference genes (Table 2 and Figure 3). Coincidentally, the two most-suited references genes were the same under each experimental condition (Figure 3). Recently, researchers have been more receptive to the use of multiple reference genes to replace a single normalizer in the RT-qPCR analysis (Vandesompele et al., 2002; Li et al., 2013; Yang et al., 2014, 2015a,b,c, 2016, 2018; Pan et al., 2015a,b), and the pairwise variations suggest that 2 reference genes were adequate for normalization under each experimental condition. Thus, *RPL13* and *RPS18* are suitable for use as the reference genes under each of the four experimental conditions.

In order to further validate the reference genes in *H. vigintioctopunctata*, the relative gene expression levels of *lov* and *TBX1* were evaluated in different tissues. Our results showed that *lov* expression was sevenfold higher in the Malpighian tubule than in the fat body when normalized to the 2 least stable reference genes *GAPDH* and *Actin* than when normalized to the 2 most stable reference ones *RPL13* and *RPS18* (Figure 5). In addition, *TBX1* expression patterns were inconsistent in the different tissues when normalized to the two best- and least-stable reference genes. The expression level was different in each tissue under the two normalization conditions (Figure 6). These results indicated that the unreasonable use of reference genes may give rise to inaccurate results for target genes. Therefore, selection and validation of the best reference genes are crucial for determining the veracity of the expression results. Thus, our study provides a more strict way to normalize RT-qPCR data in *H. vigintioctopunctata*, which will boost our comprehension of the target gene functions in this serious pest.

## Conclusion

We identified stable reference genes for RT-qPCR analysis of *H. vigintioctopunctata*. *RPL13* and *RPS18* combinations are proposed as the reference genes for each experimental condition. This study on behalf of the initial step to establish the standardized RT-qPCR analyses of *H. vigintioctopunctata* and could contribute to the in-depth functional genomic dissection of *H. vigintioctopunctata*.

## Author Contributions

HP and CY conceived and designed research. JL, SC, MG, and CY conducted experiments. BQ and JW contributed reagents. HP and JL analyzed data. HP and CY wrote the manuscript.

## Conflict of Interest Statement

The authors declare that the research was conducted in the absence of any commercial or financial relationships that could be construed as a potential conflict of interest.

## Footnotes

1 https://sg.idtdna.com/Primerquest/Home/Index

2 http://150.216.56.64/referencegene.php
