# Corrigendum: Selection and Validation of Reference Genes for RT-qPCR Analysis of the Ladybird Beetle *Henosepilachna vigintioctopunctata*

**DOI:** 10.3389/fphys.2019.00981

**Published:** 2019-07-25

**Authors:** Jing Lü, Shimin Chen, Mujuan Guo, Cuiyi Ye, Baoli Qiu, Jianhui Wu, Chunxiao Yang, Huipeng Pan

**Affiliations:** ^1^Key Laboratory of Bio-Pesticide Innovation and Application of Guangdong Province, Department of Entomology, South China Agricultural University, Guangzhou, China; ^2^State Key Laboratory for Conservation and Utilization of Subtropical Agro-Bioresources, South China Agricultural University, Guangzhou, China

**Keywords:** *Henosepilachna vigintioctopunctata*, RT-qPCR analysis, reference gene, *RefFinder*, *geNorm*

In the original article, there was an error in the title. The title “Selection and Validation of Reference Genes for RT-qPCR Analysis of the Ladybird Beetle *Henosepilachna vigintioctomaculata*” should be “Selection and Validation of Reference Genes for RT-qPCR Analysis of the Ladybird Beetle *Henosepilachna vigintioctopunctata*.”

The authors apologize for this error and state that this does not change the scientific conclusions of the article in any way.

