# Supplementary material for: Selection and Validation of Reference Genes for RT-qPCR Analysis of the Ladybird Beetle Henosepilachna vigintioctomaculata
Source: Front Physiol. 2018 Nov 14;9:1614. doi: 10.3389/fphys.2018.01614 (PMC6243213; doi:10.3389/fphys.2018.01614)
Supplement: Supplementary file 1 [file Table_1.DOCX]

**Figure S1.The agrose gel electrophoresis of these seven candidate reference genes.** M, Molecular marker; Templates in the PCR reactions were as follows: 1) *GAPDH*; 2) *Actin*; 3) *ATPB*; 4) *RPS18*; 5) *RPL6*; 6) *RPL13*, and 7) *RPL32*.

**Figure S2. Standard curves of the seven candidate reference genes.**
